# Proteomics (SWATH-MS) informed by transcriptomics approach of tropical herb *Persicaria minor* leaves upon methyl jasmonate elicitation

**DOI:** 10.7717/peerj.5525

**Published:** 2018-08-28

**Authors:** Wan Mohd Aizat, Sarah Ibrahim, Reyhaneh Rahnamaie-Tajadod, Kok-Keong Loke, Hoe-Han Goh, Normah Mohd Noor

**Affiliations:** Institute of Systems Biology (INBIOSIS), Universiti Kebangsaan Malaysia, Bangi, Selangor, Malaysia

**Keywords:** LC-MS/MS, Label-free proteomics, Methyl jasmonate, *Polygonum minus*, SWATH-MS analysis

## Abstract

**Background:**

Jasmonic acid (JA) and its derivative, methyl JA (MeJA) are hormonal cues released by plants that signal defense response to curb damages from biotic and abiotic stresses. To study such response, a tropical herbal plant, *Persicaria minor*, which possesses pungent smell and various bioactivities including antimicrobial and anticancer, was treated with MeJA. Such elicitation has been performed in hairy root cultures and plants such as Arabidopsis and rice, yet how MeJA influenced the proteome of an herbal species like *P. minor* is unknown.

**Method:**

In this study,* P. minor* plants were exogenously elicited with MeJA and leaf samples were subjected to SWATH-MS proteomics analysis. A previously published translated transcriptome database was used as a reference proteome database for a comprehensive protein sequence catalogue and to compare their differential expression.

**Results:**

From this proteomics informed by transcriptomics approach, we have successfully profiled 751 proteins of which 40 proteins were significantly different between control and MeJA-treated samples. Furthermore, a correlation analysis between both proteome and the transcriptome data sets suggests that significantly upregulated proteins were positively correlated with their cognate transcripts (Pearson’s *r* = 0.677) while a weak correlation was observed for downregulated proteins (*r* = 0.147).

**Discussion:**

MeJA treatment induced the upregulation of proteins involved in various biochemical pathways including stress response mechanism, lipid metabolism, secondary metabolite production, DNA degradation and cell wall degradation. Conversely, proteins involved in energy expensive reactions such as photosynthesis, protein synthesis and structure were significantly downregulated upon MeJA elicitation. Overall protein-transcript correlation was also weak (*r* = 0.341) suggesting the existence of post-transcriptional regulation during such stress. In conclusion, proteomics analysis using SWATH-MS analysis supplemented by the transcriptome database allows comprehensive protein profiling of this non-model herbal species upon MeJA treatment.

## Introduction

Plants are sessile and rely on hormonal cues for response against stresses. Jasmonic acid (JA) and its methylated form, methyl JA (MeJA) are among the critical hormones for such defense roles and plant adaptation. JAs are known to be induced by wounding, insects and pathogen attack, UV radiation, drought and other stresses (Dar et al., 2015). Their elicitation further invokes various cellular and molecular signalling, influencing protein expression and metabolite production to cope with stress.

*Persicaria minor* [syn. *Polygonum minus*], also known as kesum in Malaysia, is widely used as an ingredient in local cuisine due to its pungent smell. It is also used for aromatherapy and dandruff treatment (Azlim Almey et al., 2010). Several studies have reported antioxidant, antiviral, antimicrobial and anticancer activities in *P. minor* extracts (Vikram et al., 2014). Our previous results showed increase in *P. minor* leaf volatile metabolites under heat stress (Goh et al., 2016) and MeJA elicitation (Rahnamaie-Tajadod et al., 2017). Transcriptome profiling indicated the activation of defense genes involving phenylpropanoid pathway upon MeJA elicitation (Rahnamaie-Tajadod et al., 2017). However, as transcripts may be subjected to post-translational modifications, understanding MeJA signalling at the proteome level will enable key regulatory proteins involved to be identified.

Several studies have been conducted to elucidate the proteome dynamics upon MeJA elicitation in plants. However, these studies are mainly performed using Arabidopsis (Alvarez, Zhu & Chen, 2009; Chen et al., 2011), rice (Rakwal & Komatsu, 2000), as well as hairy root cultures of *Silybum marianum* (Gharechahi et al., 2013). To the best of our knowledge, no study at the proteome level of an herbal plant species such as *P. minor* has been reported on the effects of MeJA elicitation. In this study, the SWATH-MS approach was taken to quantify the proteome of control and MeJA-treated leaf samples.

SWATH-MS analysis is an emerging label-free technique in proteomics which has revolutionized many proteome research. SWATH-MS analysis is capable of profiling any given proteome quantitatively with better coverage than a traditional shotgun proteomics (which mostly relies on data-dependent acquisition) (Gillet et al., 2012). Several studies using this approach have successfully profiled proteomes from human (Bourassa et al., 2015; Chang et al., 2015), animals (Li et al., 2016; Perez-Patiño et al., 2016), microbes (Loke et al., 2016b) and plants (Zhang et al., 2016; Zhu et al., 2016). SWATH-MS proteomics is also known to be a far more sensitive, reproducible and accurate than the Western blotting alone (Gillet et al., 2012; Picotti & Aebersold, 2012). Hence, the use of SWATH-MS analysis is suited for the profiling of proteins in our non-model herbal plant.

This study aims to profile the proteome of *P. minor* leaf using SWATH-MS analysis to study the effects of MeJA treatment. As this herb is a non-model organism, a previously reported *de novo* transcriptome library (Loke et al., 2016a; Rahnamaie-Tajadod et al., 2017) was used as a reference protein sequence database, allowing a comprehensive protein profiling and identification. Furthermore, this proteome study complemented the reported transcriptome data set, allowing statistical correlation to be performed comparing their differential expression upon MeJA induced signaling. Proteins found to have significantly different abundance between control and treated samples were discussed in relation to MeJA regulation and stress physiology.

## Material & Methods

### Experimental design

**Figure 1:**
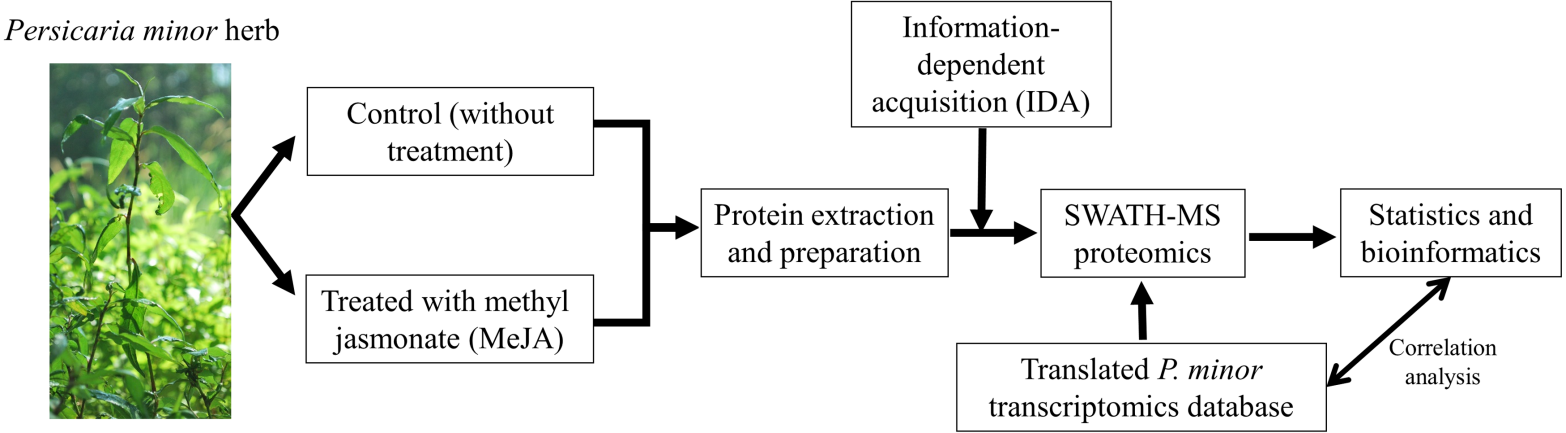
Experimental design of this study. Both control and methyl jasmonate (MeJA) treated *Persicaria minor* were prepared as described by Rahnamaie-Tajadod et al. (2017) and total proteins were extracted for subsequent proteomics analysis. A shotgun proteomics (information-dependent acquisition, IDA) was performed to generate the spectral library for the SWATH-MS analysis run (also known as data-independent acquisition, DIA). For protein identification a translated *P. minor* transcriptome database was used as the reference protein database. Both protein and transcript levels were also correlated to match their consistency. Photo credit: Normah Mohd Noor.

*P. minor* plants were treated and prepared as per Rahnamaie-Tajadod et al. (2017). The same leaf samples were utilized for both transcriptomics (Rahnamaie-Tajadod et al., 2017) and proteomics studies (this report). Total proteins were extracted from three biological replicates of control and MeJA-treated pooled leaf samples (24 h after treatment). Each biological replicate was extracted three times as three technical replicates. Overall experimental design is illustrated in Fig. 1.

### Total protein extraction

Samples (100 mg) were ground in liquid nitrogen before solubilizing in 1 mL TRI-reagent for 5 min followed by 200 µL chloroform for 15 min at room temperature (RT). Samples were then centrifuged at 2,000× g for 15 min at 4 °C and the supernatant discarded. The obtained pellets were dissolved in 300 µL 100% ethanol and incubated for 3 min at RT before centrifuged at 2,000× g for 5 min at 4 °C. The supernatants were transferred into new tubes before three times (v/v) 100% acetone were added, inverted for 15 s and incubated for 10 min at RT. Samples were then centrifuged for 12,000× g for 10 min at 4 °C and the supernatant was removed. Pellets were washed with 1 mL 0.3 M guanidine hydrochloride in 95% ethanol and 2.5% glycerol and incubated for 10 min at RT before centrifuged at 8,000× g for 5 min at RT. Resulting supernatants were discarded and the washing step was repeated twice. Then, 2.5% glycerol in absolute ethanol was added and incubated for 10 min at RT and samples were centrifuged at 8,000× g for 5 min at 4 °C. Supernatants were discarded and the pellets were dried for 10 min at RT and stored in −80 °C until required. Samples were then sent for proteomics analysis at the Australian Proteome Analysis Facility (APAF), Sydney.

### Sample preparation

The protein pellets were first solubilized in 1% sodium deoxycholate with 100 mM triethyl ammonium bicarbonate (TEAB) and agitated with five short pulses using ultrasonic probes. Samples were then heated to 95 °C for 5 min before centrifuged at 12,000× g for 5 min. Each sample was reduced with dithiothreitol (pH 9, 5 mM for 1 hr at 68 °C), then alkylated with iodoacetamide (pH 9, 12.5 mM for 1 hr at RT) and digested with trypsin overnight at 37 °C. Peptides were recovered from each sample using solid phase extraction with OMIX C18 (100 µL) tips and the eluate was dried using a vacuum concentrator. Dried peptides were resolubilized in 40 µL 2% acetonitrile and 0.1% formic acid prior to SWATH-MS analysis.

### SWATH-MS Analysis

All samples were analyzed using a TripleTOF 5600 mass spectrometer (SCIEX, Foster City, CA, USA) coupled to an Eksigent NanoLC-Ultra 2Dplus system (Eksigent Technologies, Dublin, CA, USA). Peptides were desalted using a 3.5 cm peptide trap (packed with 2.7 µm, Halo C18 solid core, 100 µm × 3.5 cm) using 2% acetonitrile (0.1% formic acid) at a flow rate of 2 µL per minute for 10 min. After desalting, the trap was switched in-line with a 20 cm C18 column (packed with 2.7 µm, Halo C18 solid core, 100 µm × 20 cm). Peptides were eluted from the column at 50 °C using a linear gradient from 95:5 mobile phase A/mobile phase B to 60:40 mobile phase A/mobile phase B (mobile phase A: 0.1% v/v formic acid; mobile phase B: 80% v/v ACN containing 0.1% v/v formic acid) over 80 min at a flow rate of 400 nL/min. All peptide separation steps were carried out at 50 °C using a column oven. The LC eluent was subjected to positive ion nanoflow analysis using an ion spray voltage, heater interface temperature, curtain gas flow and nebulizing gas flow of 2.5 kV, 150 °C, 25 and 30, respectively for the TripleTOF 5600.

Information-dependent acquisition (IDA) experiments were conducted on each biological replicate (controls and treated) using a survey scan (350–1,500 Da) of 250 millisec (ms) followed by 20 MS/MS product ion scans (350–1,500 Da) at an accumulation time of 100 ms. Product ion scans were collected for ions with a 2+ to 5+ charge-state and an ion intensity threshold of 150 counts per second (cps). Rolling collision energy (CE) was used during the IDA runs with maximum allowed CE of 80 V. SWATH experiments were conducted on each biological and technical replicate (controls and treated) using a TOF MS scan (350–1,500 Da) of 40 ms followed by 60 TOF MS/MS product ion scans in 12.5 Da windows from 400–1,150 Da each at an accumulation time of 50 ms. The collision energy was set using the CE curve (CE = 0.05 *m/z + 4) and utilised a collision energy spread of 10eV for each variable window.

### Protein identification and PeakView analysis

Proteins were identified using the Paragon search algorithm in ProteinPilot Version 4.2.0.0 (AB SCIEX, Foster City, CA, USA). A spectral library was constructed by combining searches for each of the 3 biological replicates for control and treated samples using the *P. minor* in-house transcriptome database (Loke et al., 2016a; Rahnamaie-Tajadod et al., 2017).

All SWATH files and the ProteinPilot spectral library were imported into PeakView (version 2.0.0.9257; AB SCIEX, Foster City, CA, USA). Protein level quantitation from SWATH data was performed using an extraction window of 10 min, an extracted ion chromatogram width of 10 ppm, and the following peptide filter settings: 25 peptides per protein, six transitions per peptide, 99% peptide confidence, and a 1% False Discovery Rate threshold. The mass spectrometry proteomics data was deposited to the ProteomeXchange Consortium via the PRIDE (Vizcaíno et al., 2016) partner repository with the dataset identifier PXD005749. The ProteinPilot software analysis computes the local and global False Discovery Rate (FDR) for spectral (Table S1), unique peptide (Table S2) and protein (Table S3) levels data. The peak areas for all identified proteins were incorporated in Table S4.

### Statistical analysis

**Figure 2:**
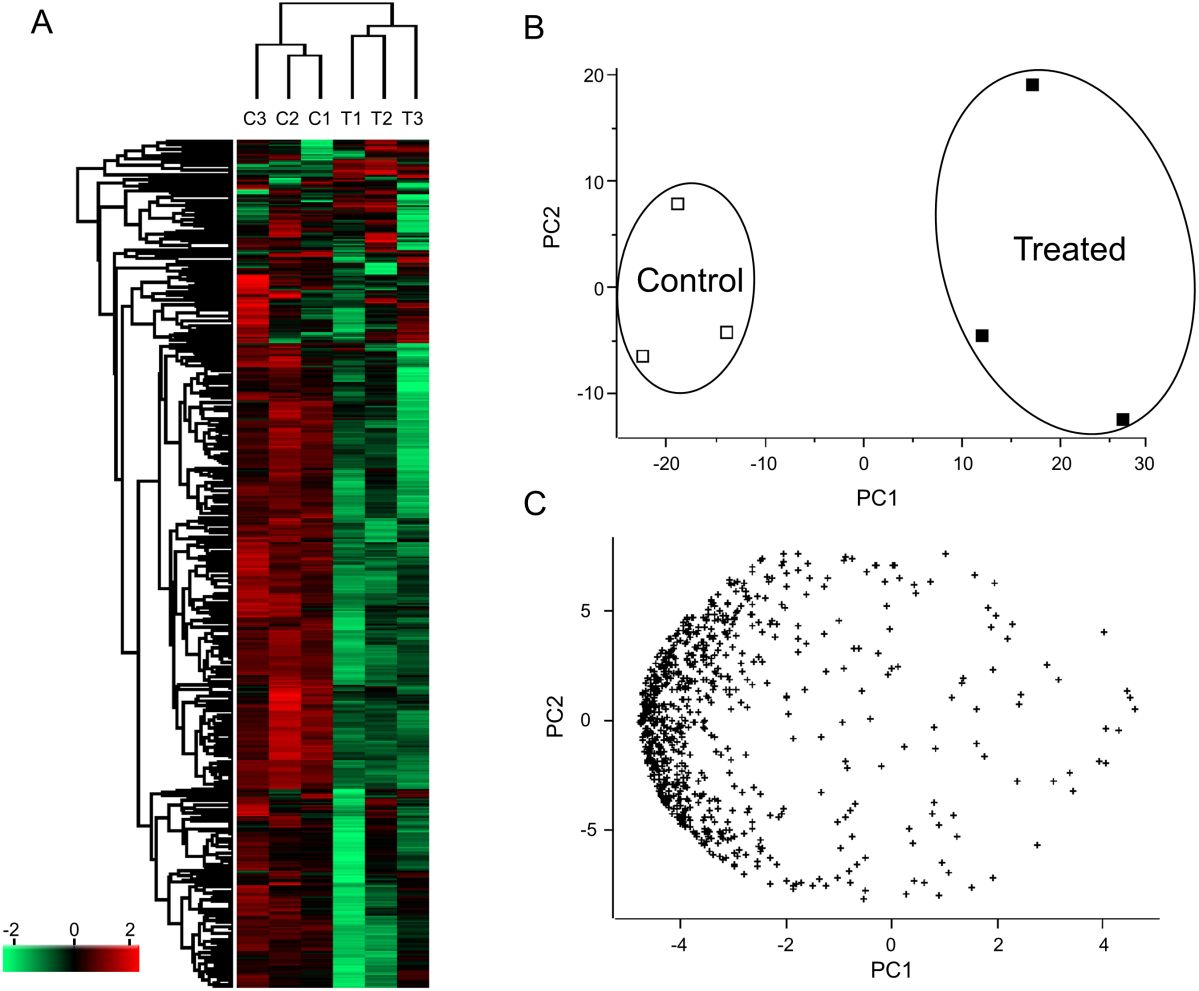
Clustering (A) and principle component analysis (B and C) of *Persicaria minor* proteome. Three biological replicates (average of two to three technical replicates each) were run for both Control (C1, C2 and C3) and Treated (T1, T2 and T3) samples. The correlation variances explained by the PC1 component is 61.2% while for PC2 component is 18.5% for both the score (B) and loading (C) plots.

All statistical analysis was performed using protein level data from PeakView and Perseus version 1.5.4.1. Peak areas were log-transformed and median-normalised to generate a normal data distribution. The data was further subjected to the principal component analysis and clustering. One technical replicate of the treated sample (T3 replicate 1) was removed from subsequent analyses for being an outlier (Aizat et al., 2018). Subsequently, the data was then normalized using z-score and clustering was performed to visualize the differences between samples via heatmap and principle component analysis (Fig. 2).

Three statistical analyses were performed to determine the differentially expressed proteins (Table S5). Firstly, a *t*-test was done to compare control and treated samples by considering all replicates (both biological and technical) as independent samples. Secondly, a *t*-test using average values of biological replicates was performed. Thirdly, the data was analyzed using a linear mixed effect model (LME) by considering a technical replicate random effect and a treatment fixed effect. Significantly differentially expressed proteins have *p* < 0.05 for all the three statistical analyses, as well as normalized fold change (FC.norm) of control over treated samples greater than 1.5 (significantly upregulated proteins) or lesser than 0.67 (significantly downregulated proteins). Comparative analysis between the proteome FC.norm and transcriptome log_2_ fold change (logFC) was performed using Pearson product moment correlation coefficient and the significance at *p*-value <0.001 was determined using a regression analysis in Microsoft Excel 2010 (Table S6).

### Bioinformatics analysis

Identified proteins were cross-referenced with transcriptome annotations as reported by Rahnamaie-Tajadod et al. (2017) for functional identification. The annotations were performed using Trinotate annotation pipeline (Loke et al., 2016a). The Blast2GO program (https://www.blast2go.com/) was also used to annotate proteins based on cellular component, molecular function and biological process (Fig. S1). This data was then graphically presented using the WEGO program (http://wego.genomics.org.cn/cgi-bin/wego/index.pl). Furthermore, pathway enrichment tool called KOBAS (http://kobas.cbi.pku.edu.cn/) was used to highlight the most enriched biological pathways in our dataset.

## Results

**Figure 3:**
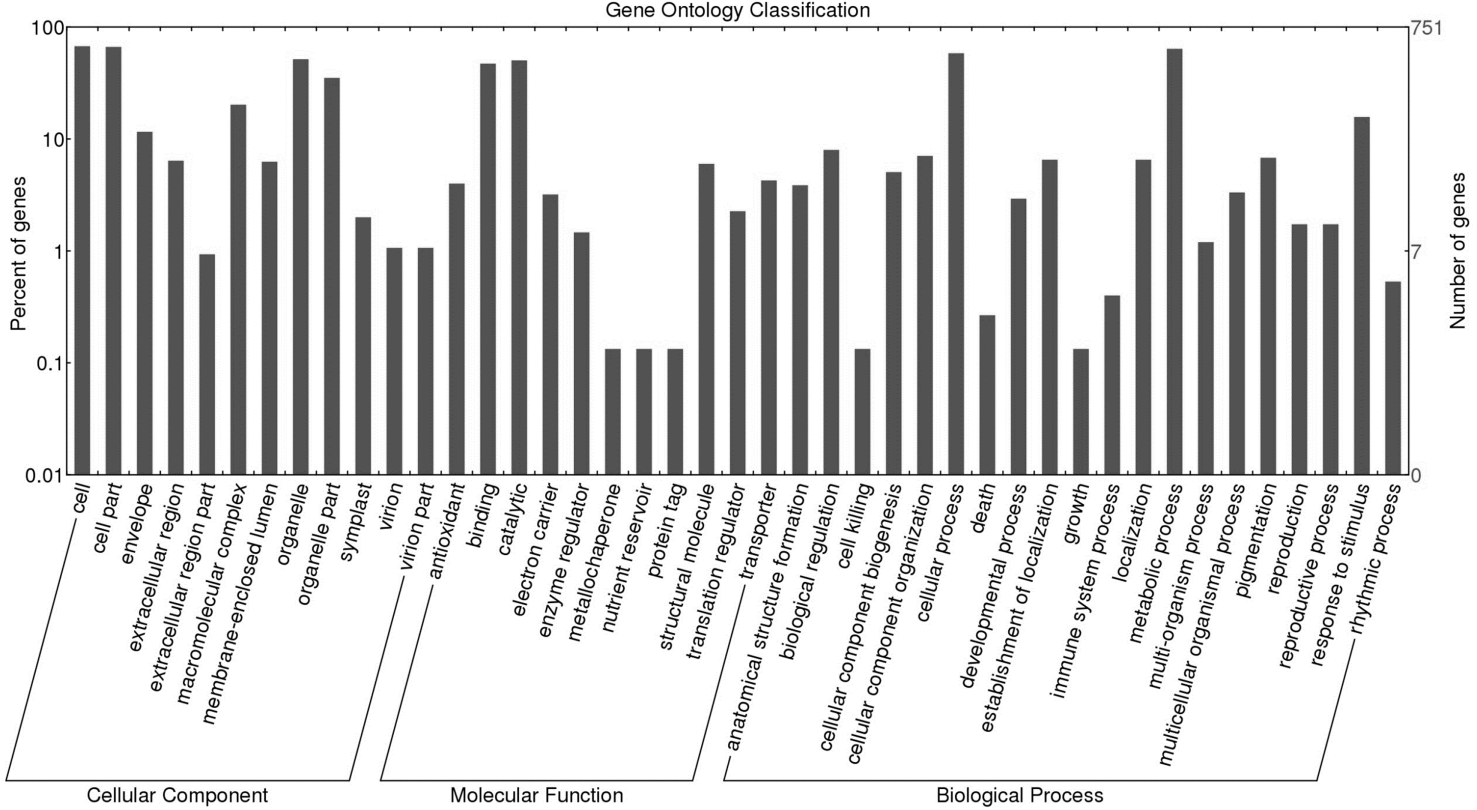
Functional classifications of *Persicaria minor* leaf proteome for all 751 identified proteins using Blast2GO software. The classification is divided into three categories: cellular component, molecular function and biological process. The percentage and number of genes are indicated on the left and right y-axis, respectively.

SWATH-MS proteomics analysis was performed to profile proteins quantitatively from *P. minor* leaf samples, which were elicited with MeJA and compared to control without treatment (Fig. 1). ProteinPilot analysis successfully identified 6,249 distinct peptides using 1% global FDR threshold (Fig. S2). Subsequently, 751 proteins were identified (Table S5 ) and they were further clustered according to either control or treated (Fig. 2). Further analysis using Blast2GO has assigned these proteins with 841 Gene Ontology (GO) terms. These terms are categorized into three different functional classifications: “cellular component”, “molecular function” and “biological process” (Fig. 3). Most protein assignments in the “cellular component” belong to “cell” (506 proteins, 67.4%), “cell part” (499, 66.4%), “organelle” (387 proteins, 51.5%) and “organelle part” (264 proteins, 35.2%). Meanwhile, “catalytic” (378 proteins, 50.3%), “binding” (354 proteins, 47.1%), “structural molecule” (45 proteins, 6.0%) and “antioxidant” (30 proteins, 4.0%) encompass the majority of the GO terms in the “molecular function”. In the “biological process” classification, the highest percentages of GO terms fall under “metabolic process” (480 proteins, 63.9%), “cellular process” (438 proteins, 58.3%), “response to stimulus” (118 proteins, 15.7%) and “biological regulation” (60 proteins, 8.0%) (Fig. 3). Most of these GO matches possessed significant *E*-value of 1 ×10^−180^ (Fig. S1A), sequence similarity distribution of more than 70% (Fig. S1B) as well as top hit species distribution to “other” species followed by beet (*Beta vulgaris*) and spinach (*Spinacia oleracea*) (Fig. S1C).

Furthermore, pathway enrichment analysis using KOBAS revealed that the top most enriched biological pathways in our proteome dataset were metabolism (corrected *p*-value of 1.79E–130), metabolic pathways (corrected *p*-value of 3.66E–85), carbon metabolism (corrected *p*-value of 3.66E–79), metabolism of proteins (corrected *p*-value of 8.00E–49) as well as metabolism of amino acids and derivatives (corrected *p*-value of 1.51E–47) (Table 1).

**Table 1:** Pathway enrichment for all identified proteins from MeJA treated *Persicaria minor* leaf using KOBAS software. Only the top five enriched pathways are listed.

**Term**	**Database**	**Input number**	**Background number**	*p*-value	Corrected *p*-value
**All identified proteins**					
Metabolism	Reactome	199	1,975	1.67E−133	1.79E−130
Metabolic pathways	KEGG pathway	133	1,243	6.83E−88	3.66E−85
Carbon metabolism	KEGG pathway	65	113	1.02E−81	3.66E−79
Metabolism of proteins	Reactome	101	1,378	2.99E−51	8.00E−49
Metabolism of amino acids and derivatives	Reactome	60	339	7.04E−50	1.51E−47

**Table 2:** Differentially expressed proteins in *Persicaria minor* leaf proteome upon methyl jasmonate (MeJA) treatment. Mean peak areas (three biological replicates) for control (Mean C) and treated (Mean T) samples were generated from SWATH-MS proteomics analysis. Differentially expressed proteins were selected based on three statistical analyses detailed in Methods (*p* < 0.05) as well as having normalized fold change (FC.norm) of control over treated samples greater than 1.5 (significantly up-regulated proteins) or lesser than 0.67 (significantly down- regulated proteins). Protein ID, protein name, UniProt accession number and organism were retrieved from the protein annotation of the *P. minor* transcriptome (Rahnamaie-Tajadod et al., 2017). Please refer to Tables S4 and  S5 for detailed descriptions of the raw data and analyses performed.

**Protein ID**	**Mean C**	**Mean T**	**FC.norm**	**Protein name**	**UniProt accession #**	**Organism**
**Significantly up-regulated proteins upon MeJA treatment**						
**Proteinase inhibitors**						
cds.c56865_g2_i9—m.103397	376,975.69	1,737,837.61	6.87	Proteinase inhibitor	P82381	*Linum usitatissimum*
cds.c56865_g2_i6—m.103394	1,555,104.16	5,873,182.80	5.58	Proteinase inhibitor	P82381	*Linum usitatissimum*
cds.c56865_g2_i3—m.103392	1,473,435.88	4,406,842.71	4.44	Proteinase inhibitor	P82381	*Linum usitatissimum*
cds.c56865_g2_i7—m.103395	2,574,250.57	3,654,504.10	2.08	Proteinase inhibitor	P82381	*Linum usitatissimum*
**Redox processes**						
cds.c55935_g2_i2—m.93522	175,025.05	668,494.17	5.37	Peroxidase 12	Q96520	*Arabidopsis thaliana*
cds.c55935_g2_i6—m.93526	1,385,492.12	1,962,003.60	2.09	Peroxidase 12	Q96520	*Arabidopsis thaliana*
cds.c51696_g2_i3—m.56033	2,223,906.61	3,067,324.59	2.03	Peroxidase 12	Q96520	*Arabidopsis thaliana*
cds.c55509_g2_i1—m.89283	2,093,087.19	2,810,777.22	2.00	Peroxidase 12	Q96520	*Arabidopsis thaliana*
cds.c48482_g2_i8—m.34843	1,360,455.24	1,524,257.70	1.63	Peroxidase 51	Q9SZE7	*Arabidopsis thaliana*
cds.c48482_g4_i2—m.34836	1,137,963.39	1,243,194.77	1.58	Peroxidase 51	Q9SZE7	*Arabidopsis thaliana*
cds.c44085_g1_i1—m.17116	98,123.59	103,360.01	1.57	Thioredoxin O1	O64764	*Arabidopsis thaliana*
**Photosynthesis**						
cds.c54051_g1_i9—m.75181	4449.63	15,270.92	5.28	Carbonic anhydrase 2	P42737	*Arabidopsis thaliana*
**Proteases**						
cds.c54084_g3_i17—m.75490	73,270.41	130,253.17	2.59	Serine carboxypeptidase-like 18;	Q9C7Z9	*Arabidopsis thaliana*
cds.c56708_g2_i3—m.101695	158,802.63	22,1382.40	2.06	Serine carboxypeptidase-like 17	Q9C7D6	*Arabidopsis thaliana*
cds.c56645_g8_i5—m.101004	305,355.00	317,549.28	1.54	Aspartic proteinase A1	O65390	*Arabidopsis thaliana*
**Lipid metabolism**						
cds.c55798_g3_i4—m.92174	46,335.46	80,368.30	2.27	Linoleate 13S-lipoxygenase 2-1	O24370	*Solanum tuberosum*
cds.c48157_g2_i2—m.33078	44,999.96	62,352.04	2.03	GDSL esterase/lipase	Q9LY84	*Arabidopsis thaliana*
**Phenolic biosynthesis**						
cds.c55964_g4_i8—m.93866	898,732.36	1,260,712.36	2.04	Flavanone-3-hydroxylase	Q06942	*Malus domestica*
cds.c48582_g2_i1—m.35349	54,305.20	58,084.49	1.59	Chorismate mutase 1	P42738	*Arabidopsis thaliana*
**Aromatic compound biosynthesis**						
cds.c56987_g1_i3—m.104679	303,398.86	329,336.50	1.54	Enone oxidoreductase	K4BW79	*Solanum lycopersicum*
**Protein glycosylation**						
cds.c50257_g2_i3—m.45689	2,777.00	3,317.73	1.86	Beta-hexosaminidase 1	A7WM73	*Arabidopsis thaliana*
**Nitrogen storage**						
cds.c51882_g2_i1—m.57255	5,854,784.07	7,322,486.75	1.84	Bark storage protein A (BSPA)	Q07469	*Populus deltoides*
**DNA binding/processing**						
cds.c46849_g1_i2—m.26698	245,864.94	294,842.61	1.83	MFP1 (Matrix Attachment Region binding filament-like protein) attachment factor 1 (MAF1)	Q9M7N6	*Solanum lycopersicum*
cds.c45380_g1_i1—m.20949	441,316.13	47,1058.03	1.62	Ubiquitin-conjugating enzyme E2 variant 1D	Q9SVD7	*Arabidopsis thaliana*
cds.c55178_g1_i9—m.85889	166,317.71	177,336.50	1.57	Endonuclease 4	F4JJL0	*Arabidopsis thaliana*
**Cell wall degradation**						
cds.c57837_g2_i2—m.114445	64,963.22	76,742.82	1.70	*α*-L-arabinofuranosidase 1 (ARAF1)	Q9SG80	*Arabidopsis thaliana*
**Cofactor biosynthesis**						
cds.c52258_g2_i5—m.60154	4,321,984.23	4,568,443.02	1.55	FO synthase	Q5YQD7	*Nocardia farcinica*
**Unknown**						
cds.c42560_g3_i2—m.13863	413,951.96	426,939.66	1.54	Uncharacterized protein	Q9ZUX4	*Arabidopsis thaliana*
**Significantly down-regulated proteins upon MeJA treatment**						
**Protein synthesis/modification**						
cds.c49616_g1_i1—m.41564	3,013,895.02	1,408,517.22	0.66	Elongation factor-Tu	P46280	*Glycine max*
cds.c44545_g1_i1—m.18392	168,891.20	54,051.02	0.44	Probable deoxyhypusine synthase	Q9YE72	*Aeropyrum pernix*
**Photosynthesis**						
cds.c56271_g1_i1—m.97063	585,856.87	265,127.66	0.66	ATP-dependent zinc metalloprotease FTSH 8	Q8W585	*Arabidopsis thaliana*
cds.c54200_g1_i2—m.76520	2,163,792.92	954,822.57	0.65	Protein translocon at the inner envelope membrane of chloroplasts (TIC) 62	Q8SKU2	*Pisum sativum*
cds.c52014_g2_i2—m.58176	585,776.63	254,375.22	0.61	chloroplast stem-loop binding protein of 41 kDa b	Q9SA52	*Arabidopsis thaliana*
cds.c52536_g1_i1—m.62408	577,417.30	176,611.11	0.48	Rhodanese-like domain-containing protein 4	Q9M158	*Arabidopsis thaliana*
cds.c54808_g4_i1—m.82256	742,278.68	228,493.84	0.45	ATP-dependent zinc metalloprotease FTSH 1	Q5Z974	*Oryza sativa*
cds.c49073_g3_i3—m.38259	748,656.47	194,635.44	0.38	Glycerate dehydrogenase	P13443	*Cucumis sativus*
**rRNA processing**						
cds.c57862_g3_i2—m.114731	199,431.98	85,355.50	0.65	Pescadillo homolog	A4RLI4	*Magnaporthe oryzae*
**Structure**						
cds.c53365_g2_i1—m.69332	383,685.96	148,936.95	0.54	Tubulin beta-2 chain	Q40106	*Lupinus albus*
**Transcription factor**						
cds.c37568_g1_i1—m.8344	102,097.61	37,178.92	0.53	Transcription factor GATA-6	P43693	*Gallus gallus*
**Unknown**						
cds.c54200_g3_i1—m.76523	399,079.08	160,138.06	0.60	Unknown protein	–	–

Among these identified proteins, 40 proteins were found to be differentially expressed between control and MeJA treated samples (Table 2) with a cut-off value of greater than 1.5-fold or lesser than 0.67-fold differences. Furthermore, these differentially expressed proteins must satisfy three statistical tests at *p*-value <0.05 as detailed in the Material and Methods section. These stringent selection criteria were employed to be selective in our discussion as well as to minimize false positive results. This is also important for our subsequent correlation analysis with the transcriptome data set.

Twenty-eight proteins were upregulated upon the treatment and these proteins were involved in various biochemical pathways and cellular processes. Four proteinase inhibitors increased between 2.08 to 6.87-folds upon the treatment whereas several peroxidases (four peroxidase 12 and two peroxidase 51) were upregulated between 1.58 to 5.37-folds. Thioredoxin O1 which is involved in the redox processes similarly as the peroxidases was also upregulated (1.57-fold). Another three proteins were proteases (serine caboxypeptidase-like 18, serine caboxypeptidase-like 17 and aspartic proteinase A1). Three other proteins were involved in DNA binding or processing such as MFP1 (Matrix Attachment Region binding filament-like protein) attachment factor 1 (MAF1), ubiquitin-conjugating enzyme E2 variant 1D and endonuclease 4 with fold changes between 1.57 to 1.83. Furthermore, other proteins involved in lipid metabolism (linoleate 13S-lipoxygenase 2-1 and GDSL esterase/lipase) as well as phenolic biosynthesis (flavanone-3-hydroxylase and chorismate mutase 1) and aromatic compound biosynthesis (enone oxidoreductase) were also differentially regulated, with 1.54 to 2.27-fold increase upon MeJA treatment. The treatment also caused the upregulation of proteins involved in photosynthesis (carbonic anhydrase 2, 5.28-fold), protein glycosylation (beta-hexominidase 1, 1.86-fold), nitrogen storage (bark storage protein A (BSPA), 1.84-fold), cell wall degradation (*α*-L-arabinofuranosidase 1, 1.7-fold), cofactor biosynthesis (FO synthase, 1.55-fold) as well as an uncharacterized protein (1.54-fold).

Twelve proteins were found to be significantly downregulated upon the MeJA treatment (Table 2). Proteins mainly involved in photosynthesis (ATP-dependent zinc metalloprotease FTSH 8, protein translocon at the inner envelope membrane of chloroplasts (TIC) 62, chloroplast stem-loop binding protein of 41 kDa b, rhodanese-like domain-containing protein 4, ATP-dependent zinc metalloprotease FTSH 1 and glycerate dehydrogenase) were downregulated by 0.38 to 0.66-folds. Whereas the expression of proteins involved in protein synthesis/modification (elongation factor-Tu and probable deoxyhypusine synthase) as well as rRNA processing (Pescadillo homolog) were also reduced by 0.44 to 0.66-folds. Moreover, other proteins involved in structural organisation (tubulin beta-2 chain, 0.54-fold), a transcription factor (transcription factor GATA-6, 0.53-fold) as well as an unknown protein (0.6-fold) were also significantly downregulated.

**Figure 4:**
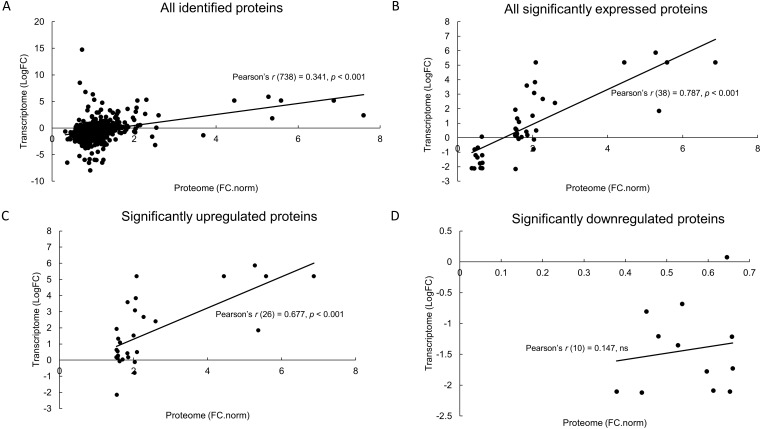
Correlation analysis between proteomics SWATH-MS data and published transcriptome RNA-seq data. Identified proteins (normalized fold change (FC.norm), this study) were compared with corresponding transcripts (Log_2_ fold change, LogFC) (Rahnamaie-Tajadod et al., 2017) using Pearson’s product moment correlation coefficient, *r*. Both datasets were compared based on either all identified proteins (A), all significantly expressed proteins (B), only significantly upregulated proteins (C) and only significantly downregulated proteins (D). Significant correlation was measured using a regression analysis at *p* < 0.001. ns, not significant.

All identified proteins were also compared with their transcript abundance from a previous transcriptomics report (Rahnamaie-Tajadod et al., 2017). Statistical correlation analysis between these proteome and transcriptome data sets suggests a weak correlation with Pearson’s *r*(738) = 0.341, *p* < 0.001 (Fig. 4A). However, a significantly positive correlation was observed for the 40 differentially expressed proteins with their corresponding transcripts (*r*(38) = 0.787, *p* < 0.001) (Fig. 4B). Among them, 28 significantly upregulated proteins also showed a strongly positive correlation (*r*(26) = 0.677, *p* < 0.001) (Fig. 4C) and the remaining downregulated proteins were weakly correlated (*r*(10) = 0.147, not significant) with their cognate transcript levels (Fig. 4D).

## Discussion

Proteomics study relies heavily on the protein sequence database which is often lacking in non-model species such as herbs including *P. minor.* The use of integrative proteomics and transcriptomics approaches (Fig. 1) allow a comprehensive study on this herb which successfully identified 751 proteins from the SWATH-MS analysis. MeJA elicitation also has been known to trigger various responses and this is further substantiated by current findings that a rather high percentage (15.7%) of identified proteins was assigned to the “response to stimulus” GO term (Fig. 3). Furthermore, “cellular process”, “metabolic process” and “biological regulation” terms were also having the most protein assignment (Fig. 3), suggesting that biochemical processes might be actively regulated in the proteome of *P. minor* in response to MeJA. Evidently, the differentially expressed proteins (Table 2) further indicate that several key biological pathways were modulated, including stress response mechanism, lipid metabolism, secondary metabolite production, apoptosis, photosynthesis as well as the regulation of protein synthesis and structural proteins.

### MeJA triggers proteins related to stress response mechanism, lipid metabolism and secondary metabolite production

Several proteins related to defense and stress responses were differentially regulated upon the MeJA elicitation. For example, four proteinase inhibitors were significantly upregulated. These proteins were shown to be activated by similar elicitation in tomato, alfalfa, tobacco (Bolter & Jongsma, 1995; Farmer & Ryan, 1992) and maize (Zhang et al., 2015), mimicking a wounding response. These proteinase inhibitors are important for plant defense responses, capable of disrupting digestive system of insects to deter them from feeding on the plants (Sharma, 2015). Furthermore, an enzyme responsible for protein glycosylation, beta-hexominidase has also been implicated in plant defense against insects as it is shown to possess chitinase activity (Strasser et al., 2007). Another wound inducible protein with potential chitinase activity is BSPA (Meuriot et al., 2004). This protein was also implicated in nitrogen storage (Meuriot et al., 2004), yet its upregulation together with beta-hexominidase after MeJA treatment may imply the activation of a defense mechanism against such stress signal.

Stresses such as wounding also caused plants to generate reactive oxygen species (ROS). ROS may attack cellular proteins and enzymes causing aberrant modifications to their structural integrity and functions (Ray, Huang & Tsuji, 2012). Therefore, the presence of proteases during stress conditions are crucial for protein homeostasis. Our proteomics study revealed significant upregulation of a few proteases such as serine carboxypeptide-like 17, serine carboxypeptide-like 18 and aspartic proteinase A1 upon MeJA treatment. Consistent with our report, these proteins were also stimulated upon injury and MeJA treatment in other plant species such as tomato and rice (Feng & Xue, 2006; Moura, Bergey & Ryan, 2001).

Given that ROS are damaging to the plant cells, their homeostasis through redox-related enzymes such as peroxidases and thioredoxin are important for cellular integrity and biochemical processes (Zhang et al., 2015). In this study, several peroxidases (four peroxidases 12 and two peroxidases 51) and one thioredoxin (thioredoxin O1) were upregulated upon exogenous application of MeJA, suggesting their involvement in stress response. Peroxidases were also involved in lignin and suberin polymerization (Singh, Rastogi & Dwivedi, 2010), perhaps providing extracellular structural strength during an attack. Another upregulated protein, FO synthase which catalyses the coenzyme FO (7,8-didemethyl-8-hydroxy-5-deazariboflavin) may also be involved in this redox-related process (Grochowski, Xu & White, 2009) upon MeJA elicitation.

Furthermore, lipid metabolism may also be induced by exogenous MeJA application. Two lipolytic enzymes, linoleate 13S-lipoxygenase 2-1 and GDSL esterase/lipase were upregulated more than 2-fold upon the treatment. Interestingly, linoleate 13S-lipoxygenase 2-1 is the first enzyme in the α-linolenic acid metabolic pathway producing jasmonate (Luge et al., 2014). Proteomics study of *Nicotiana occidentalis* infected with phytoplasma, a pathogenic bacterium also revealed significant increase in both linoleate 13S-lipoxygenase 2-1 enzyme and jasmonate level (Luge et al., 2014). This suggests that the enzyme regulates positive feedbacks of jasmonate production during an injury response or stress. Furthermore, *GER1* gene (GDSL containing enzyme rice 1) containing a GDSL motif was responsible for the light regulated JA response (Riemann et al., 2007), suggesting that the GDSL esterase/lipase could also be part of the defense mechanism mediated by jasmonate (Chepyshko et al., 2012).

Application of MeJA has also been known to induce secondary metabolite production including phenolic compounds such as flavonoids (Petrussa et al., 2013). Enzymes involved in this phenolic pathway, chorismate mutase and flavanone-3-hydroxylase (Tohge et al., 2013) were significantly upregulated in the MeJA treated herbs. Phenolics increase is associated with defense mechanism, owing to its antimicrobial activity to curb attacks from microbes during an injury response (Petrussa et al., 2013). Another secondary metabolite biosynthetic protein, enone oxidoreductase is involved in the production of furaneol, an aromatic volatile compound found in fruit such as pineapple and strawberry (Eshghi, Jamali & Rowshan, 2014; Klein et al., 2007). *P. minor* has been shown to increase both flavonoid and volatile organic compounds upon high temperature treatment (Goh et al., 2016), and this study further implies that MeJA elicitation could also exhibit similar response.

### MeJA treatment induced apoptosis related proteins and downregulated photosynthetic proteins

Significant stresses on plants including biotic and abiotic factors may induce cellular apoptosis which can be characterized by hallmark events such as DNA degradation (Kacprzyk, Daly & McCabe, 2011; Reape & McCabe, 2008). In this study, a protein potentially involved in this event, endonuclease 4 (Triques et al., 2007) was significantly upregulated in MeJA treated samples. In a tobacco plant infected with a tobacco mosaic virus, nucleases activities were induced to degrade DNA (Mittler, Simon & Lam, 1997). This is perhaps as a means of recycling building blocks of the DNA during programmed cell death after pathogen invasion or stress signals (Reape & McCabe, 2008). Interestingly, ubiquitin-conjugating enzyme E2 variant 1D protein involved in the DNA damage tolerance (Wen et al., 2008) was also upregulated, suggesting DNA repair mechanism might be activated almost simultaneously upon DNA degradation. Despite such repair, the increase of endonuclease 4 as early as 24 h after MeJA treatment can be detrimental to the plant. Our study indicates that MeJA signal may induce apoptotic events such as leaf decoloration (due to chlorophyll breakdown) and yellowing towards our *P. minor* plants after 3 to 5 days upon treatment (Rahnamaie-Tajadod et al., 2017). This further suggests that photosynthesis and chloroplast integrity might be affected by the MeJA elicitation.

Congruently, several proteins involved in photosynthesis and/or chloroplast components such as ATP-dependent zinc metalloprotease FTSH 1 and 8, rhodanese-like domain containing protein 4, glycerate dehydrogenase, chloroplast stem-loop binding protein of 41 kDa b as well as protein TIC62 were significantly downregulated in *P. minor* leaf treated with MeJA. This suggests that in response to stress, plants reduced energy expensive processes such as photosynthesis, saving the energy for its defense mechanism (Attaran et al., 2014). In other species such as rice (Rakwal & Komatsu, 2000) and Arabidopsis (Chen et al., 2011), some photosynthetic proteins such as ribulose-1,5-bisphosphate carboxylase/oxygenase (RuBisCO) were also reduced upon JA and MeJA treatment, respectively. This implies similar downregulation response towards photosynthesis upon exposure to stress signals in various plants. Interestingly, the level of carbonic anhydrase 2 involved in photosynthesis was significantly increased (more than 5-fold) in our *P. minor* treated samples. Carbonic anhydrase was known to be involved in salt stress adaptation and JA treatment caused its upregulation (Walia et al., 2007). MeJA signals might upregulate its expression in this study, but the main reason why such photosynthetic protein increased after the stress signals remains to be investigated.

Another protein found to be upregulated upon MeJA elicitation was MAF1. It is a nuclear structural protein responsible for the attachment of chromatin to the nuclear matrix, with other interacting partners (Gindullis, Peffer & Meier, 1999). MAF1 has also been shown to be significantly increased in drought-induced wheat (Bazargani et al., 2011), suggesting its potential role in chromatin reorganization during stress. Interestingly, another characteristic event for apoptosis is chromatin condensation (Reape & McCabe, 2008), but whether MAF1 also involved in this process requires further study.

### MeJA negatively regulates protein synthesis and structural proteins

Proteins involved in the synthesis and modification of proteins such as elongation factor-Tu (EF-Tu) (Fu, Momčilović & Prasad, 2012) and probable deoxyhypusine synthase (Park et al., 2010) respectively, were decreased upon MeJA elicitation (Table 2). Furthermore, a protein related to rRNA processing, Pescadillo homolog protein (Zografidis et al., 2014) as well as a transcription factor GATA-6 were also downregulated. This implies that protein synthesis was switched off during an injury response induced by MeJA. This further supports plant strategy for energy conservation during an attack or stress signals, to cater for defense and recovery.

MeJA induction may also influence plant growth and cell wall structure. In this study, a structural protein, tubulin beta-2 chain was downregulated whereas a cell wall remodelling protein, α-L-arabinofuranosidase 1 (ARAF1) was upregulated. Beta-tubulin, a component of microtubules in cells is mainly involved in plant cell division, elongation and cell wall structure formation (Yakovleva, Egorova & Tarchevsky, 2013). Tubulin has also been shown to be downregulated, along with plant growth impediment in rice (Cho et al., 2007) and pea (Yakovleva, Egorova & Tarchevsky, 2013) upon JA or MeJA elicitation, respectively. Furthermore, ARAF1 is associated with polysaccharide degradation in plant cell wall (Minic et al., 2004), suggesting its upregulation upon stress signal may contribute to cell wall modification. This further supports that growth-related processes are suppressed in plant treated with MeJA, as would be expected in a stress condition, perhaps in response to the apoptosis discussed earlier.

Two unknown proteins were also found to be either upregulated or downregulated, respectively upon MeJA treatment (Table 2). As such, these proteins may be associated with stress conditions, yet further investigation are needed to characterize their functions and roles in this *P. minor* herbs.

### Correlation analysis between proteome and transcriptome data reveals the existence of post-transcriptional and post-translational regulation

In the previous transcriptome study, several candidate genes involved in jasmonate biosynthesis and signaling were validated using quantitative Real Time-PCR (qRT-PCR) (Rahnamaie-Tajadod et al., 2017). The results showed a considerably good correlation (*R*^2^ = 0.82) between the RNAseq and qRT-PCR data. In this study, we have extended such analysis to compare both the transcriptome and currently obtained proteome data. As this plant is considered as a non-model organism, we have used the transcriptome non-redundant sequences as our reference sequence database for protein identification (proteomics informed by transcriptomics approach) and as such comparative analysis is important to illuminate complementing or contrasting trends between the different molecular levels, proteins and transcripts. Other plant proteomics studies have only utilised qRT-PCR for complementing their protein expression levels such as in Botrytis infected kiwifruit (Liu et al., 2018), virus infected rice (Yu et al., 2017) and rapeseed varieties (Wang et al., 2018). The comparison between both proteome and transcriptome as performed in this report will allow even wider comparative analysis of the expression profiles between these two omics studies.

Our result suggests that the majority of both proteome and transcriptome expression levels do not correlate as measured by Pearson’s *r* = 0.341 (Fig. 4A), differences that can be attributed to the existence of post-transcriptional regulation. Such regulation is common in eukaryotic organisms as shown previously in the omics studies of plant nematodes (Zhou et al., 2016) and the stress treatments of Arabidopsis (Lan, Li & Schmidt, 2012; Liang et al., 2016; Marmiroli et al., 2015). Interestingly, a comparison between significantly expressed proteins and their corresponding transcripts were positively well-correlated (*r* = 0.787) (Fig. 4B). Such significant correlation may have been mostly attributed by the proteins that were upregulated (*r* = 0.677) (Fig. 4C) rather than the downregulated proteins (*r* = 0.147) (Fig. 4D). These contrasting correlations were similarly observed in phosphate deficient Arabidopsis (Lan, Li & Schmidt, 2012), speculating that stresses may specifically induce protein degradation post-translationally while not affecting transcript levels. This may serve as an advantage to the plant, as further *de novo* transcription is not needed if the stress is released (Lan, Li & Schmidt, 2012). Another possibility is that transcript and protein downregulation may have different timing such that their monitoring at a specific time point is not possible (Lan, Li & Schmidt, 2012). However, further experimentation is needed to account for these notions.

## Conclusions

In conclusion, this is the first proteomics study on this non-model herbal species, *P. minor* particularly using an advance proteomics platform called SWATH-MS, enriched with a previously reported transcriptome database for protein identification. This has enabled us to profile a comprehensive proteome coverage of 751 proteins, and forty were found differentially expressed. The modulated levels of these proteins suggest that the hormone invoked defense and recovery response but suppressed proteins involved in growth and development. Correlation analysis between our proteome study and previously reported transcriptome analysis suggests that post-transcriptional and post-translational regulation may have existed to regulate certain groups of proteins.

##  Supplemental Information

10.7717/peerj.5525/supp-1 Figure S1 Basic bioinformatics obtained through Blast2GO analysis which include *E*-value distribution, sequence similarity distribution and top-hit species distribution

10.7717/peerj.5525/supp-2 Figure S2 Distinct peptide level False Discovery Rate (FDR) analysis using ProteinPilot software

10.7717/peerj.5525/supp-3 Table S1 Spectral level data from ProteinPilot software

10.7717/peerj.5525/supp-4 Table S2 Distinct peptide level data from ProteinPilot software

10.7717/peerj.5525/supp-5 Table S3 Protein level data from ProteinPilot software

10.7717/peerj.5525/supp-6 Table S4 The raw data for the peak areas of 751 identified proteins from *Persicaria minor* Three biological replicates for both control (C1, C2 and C3) and treated (T1, T2 and T3) samples as well as three technical replicates (rep 1, rep 2 and rep 3) for each biological replicate were run using SWATH-MS analysis.

10.7717/peerj.5525/supp-7 Table S5 The statistical analyses performed for the 751 identified proteins from *Persicaria minor* Mean peak areas (three biological replicates) for control (Mean C) and treated (Mean T) samples were generated from SWATH-MS proteomics analysis.

10.7717/peerj.5525/supp-8 Table S6 Raw data of the correlation between proteomics and transcriptomics results The data were correlated using Pearson product moment correlation coefficient, *r*.

## Additional Information and Declarations

### Data Availability

The following information was supplied regarding data availability: ProteomeXhange Dataset identifier PXD005749.
