# Supplementary figures and images for: Proteomics (SWATH-MS) informed by transcriptomics approach of tropical herb Persicaria minor leaves upon methyl jasmonate elicitation
Source: PeerJ. 2018 Aug 28;6:e5525. doi: 10.7717/peerj.5525 (PMC6118203; doi:10.7717/peerj.5525)

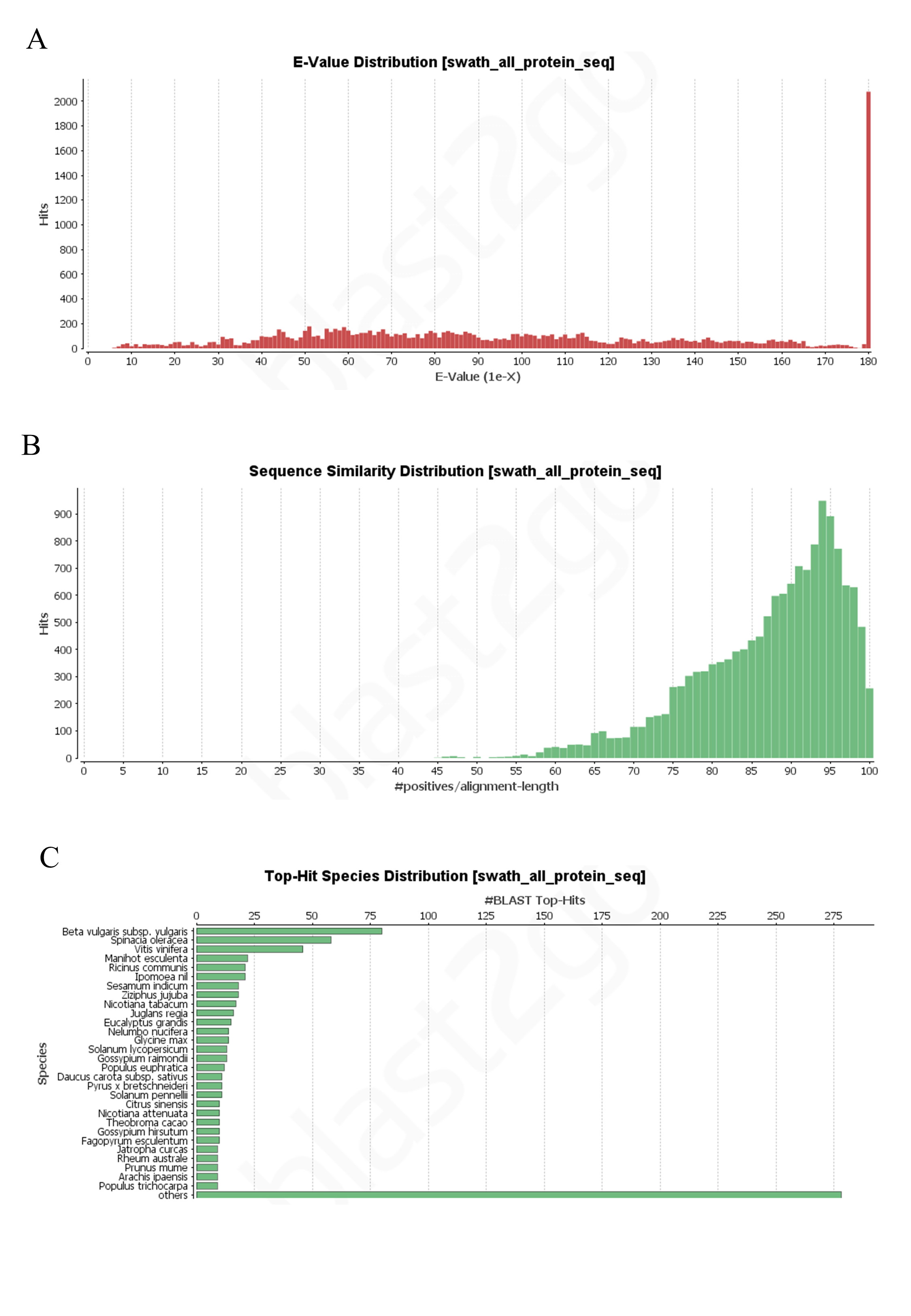

Supplement: Figure S1 [file peerj-06-5525-s001.png]

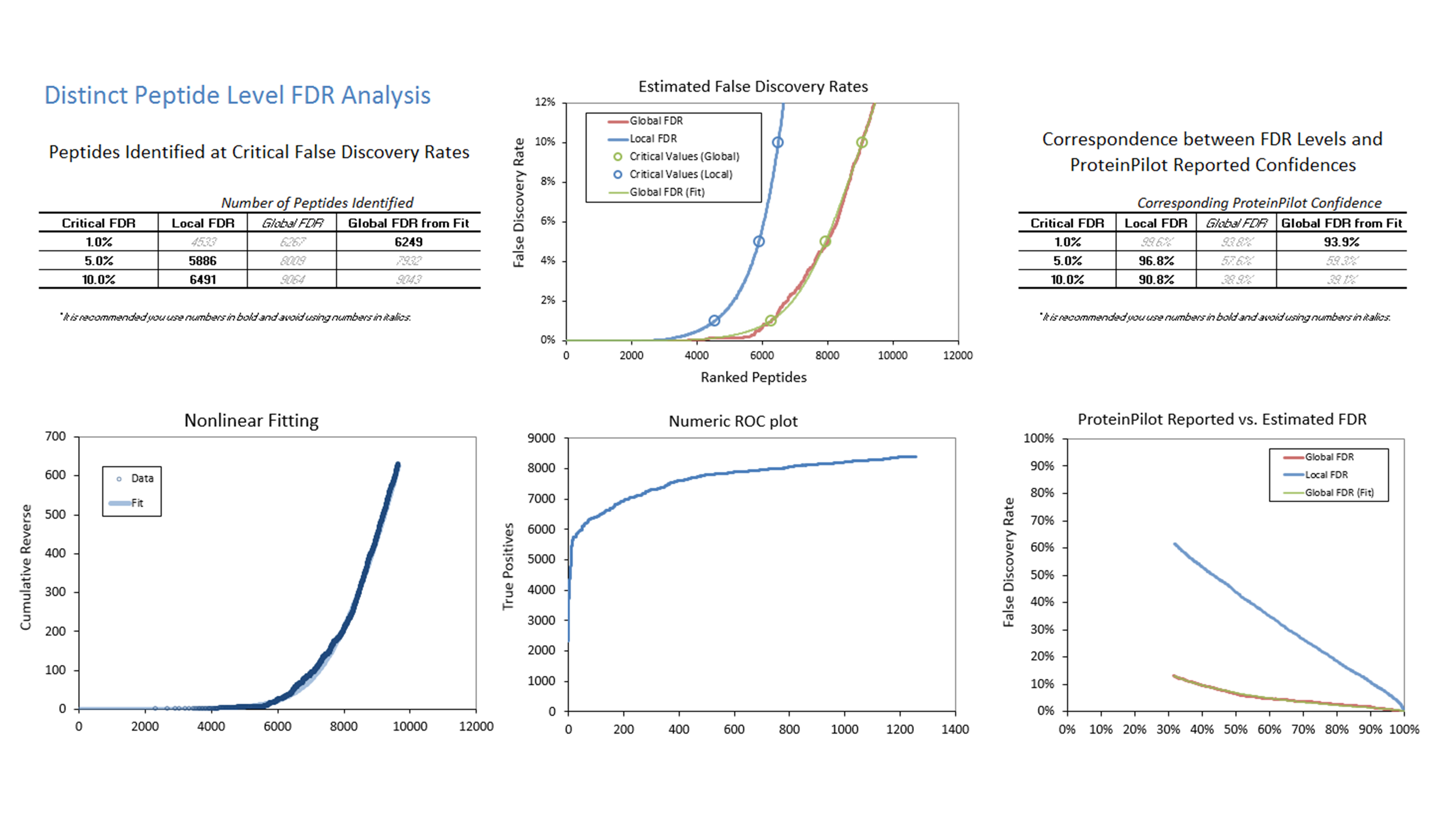

Supplement: Figure S2 [file peerj-06-5525-s002.png]
